# Supplementary material for: Maximal information transmission is compatible with ultrasensitive biological pathways
Source: Sci Rep. 2019 Nov 15;9:16898. doi: 10.1038/s41598-019-53273-4 (PMC6858467; doi:10.1038/s41598-019-53273-4)
Supplement: Supplementary file 1 — Supplementary information [file 41598_2019_53273_MOESM1_ESM.pdf]

Supporting information: Maximal information transmission is compatible with ultrasensitive biological pathways

Gabriele Micali[1,2,3,4], Robert G. Endres[1,2,†]
1 Department of Life Sciences, Imperial College, London, UK
2 Centre for Integrative Systems Biology and Bioinformatics at Imperial College, London, UK
3 Department of Environmental Microbiology, Eawag, Dübendorf, Switzerland
4 Department of Environmental Systems Science, ETH Zürich, Zürich, Switzerland
† E-mail: r.endres@imperial.ac.uk

Contents

1 Mathematical details on maximizing mutual information with respect to both input distribution and input-output curve 2
1.1 Maximization with respect to the input distribution . . . 3
1.2 Maximization with respect to the input-output curve . . . 3
1.3 Comparison of the output distributions between the two maximization approaches . . . 4
1.4 Uniform noise . . . 4
1.5 Input noise only . . . 4
1.6 Output noise only . . . 5
1.7 Independent transmitted input and output noise . . . 5
1.7.1 Noise: $\sigma_{\mathrm{T}}(x, \bar{y}(x), \bar{y}'(x)) = \sqrt{\alpha_1 x \bar{y}'^2 + \alpha_4}$, with constants $\alpha_{1,4}$ . . . 5
1.7.2 Noise: $\sigma_{\mathrm{T}}(x, \bar{y}(x), \bar{y}'(x)) = \sqrt{\alpha_1 x \bar{y}'^2 + \alpha_3 \bar{y} + \alpha_4}$, with constants $\alpha_{1,3,4}$ . . . 6
1.7.3 Noise: $\sigma_{\mathrm{T}}(x, \bar{y}(x), \bar{y}'(x)) = \sqrt{\alpha_1 x \bar{y}'^2 + \alpha_2 \bar{y}(1-\bar{y}) + \alpha_3 \bar{y} + \alpha_4}$, with constants α_{1-4} 6
1.7.4 Boundary conditions on $\bar{y}'$ that produce smooth input-output curves . . . 7
1.7.5 Inverse problem: predicting internal noise for an optimal Hill-function solution . . 7
1.7.6 The optimal input distribution only depends on the input noise . . . 8
1.8 Discussion of the sensitive region . . . 8

2 Mutual information and Fisher information 8

3 Sources of noise and estimation of noise parameters 10

4 Additional results 11
4.1 Optimal information transmission for uniform noise . . . 11
4.2 Noise sensitivity . . . 11
4.2.1 Joint optimization for different α values . . . 11
4.2.2 Total noise for optimal information transmission for different types of output noise 12
4.2.3 Comparison of the total noise at the receptor and motor channels . . . 12
4.2.4 High information transmission for multiple motors with high Hill coefficients is robust to changes in noise level . . . 12
4.3 Comparison of optimal input-output curves with experimental data from [1] . . . 12
4.4 Mutual information between $\mathrm{CheY_p}$ and motor bias for multiple motors . . . 13
4.4.1 Maximization of information transmission for the two-motor channel . . . 15
4.5 Estimating the mutual information between the external ligand concentration and motor bias . . . 15
4.5.1 Mutual information when the motor noise is much smaller than the receptor noise 15
4.5.2 Mutual information when the receptor noise is much smaller than the motor noise 16
4.5.3 Numerical approach . . . 16

4.5.4 The effect of the motor threshold k_d^m on information transmission 17
4.6 Test of conditional independent motors 17

1 Mathematical details on maximizing mutual information with respect to both input distribution and input-output curve

Mutual information has been introduced by Shannon to quantify the dependencies between inputs and outputs of a communication channel [2]. Among all possible measures of correlations, the mutual information has many properties which are useful when the theory is generalized to biological channels [3–6]. For continuous random variables, the mutual information is defined by

$$\mathcal{I}[X,Y] := \int dx\ dy\ p(x,y)\log_2\left[\frac{p(x,y)}{p(x)p(y)}\right] = \int dx\ dy\ p(x,y)\log_2\left[\frac{p(y|x)}{p(y)}\right], \tag{S1}$$

where $p(x,y)$ is the joint probability of observing $X=x$ and $Y=y$, and $p(y|x)$ is the conditional probability of observing y given x. By rewriting the logarithms we obtain,

$$\mathcal{I}[X,Y] = \int dx\, p(x)\int dy\ p(y|x)\log_2 p(y|x) - \int dx\, p(x)\int dy\ p(y|x)\log_2 p(y), \tag{S2}$$

where we used $p(x,y)=p(y|x)p(x)$. Assuming a Gaussian channel, i.e.

$$p(y|x) = \frac{1}{\sqrt{2\pi\sigma_{\mathrm{T}}^2}}\exp\left[-\frac{(y-\bar{y})^2}{2\sigma_{\mathrm{T}}^2}\right] \tag{S3}$$

with mean $\bar{y}(x)$ and standard deviation $\sigma_{\mathrm{T}}(x)$, the first term on the right-hand side of Eq. (S2) is the entropy of a Gaussian (with a minus sign) and Eq. (S2) becomes

$$\mathcal{I}[X,Y] = -\int dx\, p(x)\log_2\sqrt{2\pi \mathrm{e}}\sigma_{\mathrm{T}}(x) - \int dx\, p(x)\int dy\ p(y|x)\log_2 p(y). \tag{S4}$$

Furthermore, in the small-noise approximation we write $\int dy\ p(y|x)\log_2 p(y) \approx \log_2 p(\bar{y})$. Finally, by using conservation of probability $p(\bar{y})d\bar{y} = p(x)dx$, Eq. (S4) becomes

$$\mathcal{I}[X,Y] = -\int dx\, p(x)\log_2\frac{\sqrt{2\pi\mathrm{e}}\sigma_{\mathrm{T}}(x)p(x)}{\bar{y}'(x)}, \tag{S5}$$

which is identical to Eq. (2) in the main text [7–11].

To maximize Eq. (S5), we focus on the integrand, i.e. the Lagrangian

$$\mathcal{L}(x,p,\bar{y},\bar{y}') = -p(x)\log_2\left[\frac{\sqrt{2\pi e}\sigma_{\mathrm{T}}(x,\bar{y}(x),\bar{y}'(x))}{\bar{y}'(x)}p(x)\right], \tag{S6}$$

and apply the Euler-Lagrange equations, where x is the independent variable and $\bar{y}=\bar{y}(x)$, $\bar{y}'=\bar{y}'(x)$ and $p=p(x)$ are the dependent variables. This leads to a system of two equations,

$$\begin{cases} \frac{\partial\mathcal{L}}{\partial p} = 0, \\ \frac{\partial\mathcal{L}}{\partial\bar{y}} = \frac{d}{dx}\frac{\partial\mathcal{L}}{\partial\bar{y}'}, \end{cases} \tag{S7}$$

where the first equation is the maximization with respect to the input distribution p and the second equation is the maximization with respect to the input-output curve $\bar{y}$. In the following, we first solve these two equations separately. Subsequently, we solve the system of equations simultaneously for different choices of noise $\sigma_{\mathrm{T}}(x,\bar{y}(x),\bar{y}'(x))$.

1.1 Maximization with respect to the input distribution

To maximize the mutual information, we can maximize $\mathcal{L}(x, p, \bar{y}, \bar{y}')$ in Eq. (S6) with respect to the input probability p given the constraint of normalization,

$$\frac{\partial}{\partial p}\left[\int \mathcal{L}\ \mathrm{d}x - \Lambda\left(\int p\ \mathrm{d}x - 1\right)\right] = 0\ , \tag{S8}$$

which is solved by $\log_2\left[\frac{\sqrt{2\pi e}\sigma_\mathrm{T}}{\bar{y}'}p\right] + 1 - \Lambda = 0$, and hence results in the input distribution

$$p(x) = \frac{\bar{y}'(x)(\Lambda - 1)}{\sqrt{2\pi e}\sigma_\mathrm{T}(x)} = \frac{\bar{y}'(x)}{Z\sigma_\mathrm{T}(x)} \tag{S9}$$

with $Z = \sqrt{2\pi e}/(\Lambda - 1)$ a normalization factor.

1.2 Maximization with respect to the input-output curve

To maximize with respect to the input-output curve, we follow our work in [11]. The equation we need to solve is

$$\frac{\partial \mathcal{L}}{\partial \bar{y}} - \frac{\mathrm{d}}{\mathrm{d}x}\frac{\partial \mathcal{L}}{\partial \bar{y}'} = 0\ , \tag{S10}$$

with $\mathcal{L}(x, p, \bar{y}, \bar{y}')$ from Eq. (S6). Assuming independent transmitted input and output noise, i.e. $\sigma_\mathrm{T}^2 = \sigma_\mathrm{X}^2(\bar{y}')^2 + \sigma_\mathrm{Y}^2$, we observe that

$$\frac{\partial \mathcal{L}}{\partial \bar{y}} = \frac{p}{\sigma_\mathrm{T}}\frac{\partial \sigma_\mathrm{T}}{\partial \bar{y}} = \frac{p}{2\sigma_\mathrm{T}^2}\frac{\partial \sigma_\mathrm{Y}^2}{\partial \bar{y}}, \tag{S11}$$

$$\frac{\partial \mathcal{L}}{\partial \bar{y}'} = \frac{p}{\sigma_\mathrm{T}}\frac{\partial \sigma_\mathrm{T}}{\partial \bar{y}'} - \frac{p}{\bar{y}'} = -\frac{p\sigma_\mathrm{Y}^2}{\bar{y}'\sigma_\mathrm{T}^2}, \tag{S12}$$

$$\frac{d}{dx}\frac{\partial \mathcal{L}}{\partial \bar{y}'} = -\frac{p'}{\bar{y}'} + \frac{p\bar{y}''}{\bar{y}'^2} - \frac{p'}{\sigma_\mathrm{T}}\frac{\partial \sigma_\mathrm{T}}{\partial \bar{y}'} + \frac{p\sigma_\mathrm{T}'}{\sigma_\mathrm{T}^2}\frac{\partial \sigma_\mathrm{T}}{\partial \bar{y}} + \frac{p}{\sigma_\mathrm{T}}\frac{d}{dx}\frac{\partial \sigma_\mathrm{T}}{\partial \bar{y}'} = -\xi p' - p\xi', \tag{S13}$$

where $\xi = \frac{\sigma_\mathrm{Y}^2}{\bar{y}'\sigma_\mathrm{T}^2}$. Eq. (S10) then becomes

$$-\frac{\xi'}{\xi} = \frac{\bar{y}'}{2\sigma_\mathrm{Y}^2}\frac{\partial \sigma_\mathrm{Y}^2}{\partial \bar{y}} + \frac{p'}{p}\ . \tag{S14}$$

Integrating this equation in x gives

$$\xi p \sigma_\mathrm{Y} = \frac{\sigma_\mathrm{Y}^3 p}{\bar{y}'\sigma_\mathrm{T}^2} = K\ , \tag{S15}$$

where K is set by boundary conditions. By integrating this equation once more, we can get the optimal input-output curve. In addition, we also observe that by performing this optimization we obtain $p \propto \frac{\bar{y}'\sigma_\mathrm{T}^2}{\sigma_\mathrm{Y}^3}$. Before moving to the joint maximization we discuss the differences between the output distributions of the two different approaches.

1.3 Comparison of the output distributions between the two maximization approaches

The output distribution should in principle be calculated by

$$p(y) = \int p(y|x)p(x)\ \mathrm{d}x \approx p(x)/\bar{y}'\ , \tag{S16}$$

where the approximation is valid only in the small noise regime, following from the conservation of the probability measure $p(y)\mathrm{d}y = p(x)\mathrm{d}x$. For the two different approaches, we then obtain

$$p(y)|_p = \frac{1}{Z\sigma_{\mathrm{T}}(y)} \qquad \text{maximization with respect to } p \tag{S17}$$

$$p(y)|_{\bar{y}} = \frac{\sigma_{\mathrm{T}}^2(y)}{W\sigma_{\mathrm{Y}}^3(y)} \qquad \text{maximization with respect to } \bar{y} \tag{S18}$$

with Z and W normalization factors. It is important to notice that for small transmitted input noise $p(y)|_{\bar{y}} \approx \frac{1}{W\sigma_{\mathrm{Y}}(y)} = p(y)|_p$, while for large transmitted input noise $p(y)|_{\bar{y}} \approx \frac{(\bar{y}')^2\sigma_{\mathrm{X}}^2(y)}{W\sigma_{\mathrm{Y}}^3(y)} \neq \frac{1}{Z\sigma_{\mathrm{T}}(y)} = p(y)|_p$.

The output distribution can be bimodal or unimodal, depending on the shape of the noise. The transmitted input noise has a peak when the gain is high, i.e. high $\bar{y}'$, while the output noise might also have a peak in the same region due to switching noise. Hence, the output distribution for the maximization with respect to the input distribution is expected to be bimodal under such conditions. In contrast, the maximization with respect to the input-output distribution lead to bimodality when $\sigma_{\mathrm{T}}^2(y) > \sigma_{\mathrm{Y}}^3(y)$ for intermediate values of y and $\sigma_{\mathrm{T}}^2(y) < \sigma_{\mathrm{Y}}^3(y)$ close to the boundary of y (see Fig. 2 in the main text). The two maximizations will behave similarly for negligible transmitted input noise. In the following, we solve the system of equations in Eq. (S7) for different choices of noise $\sigma_{\mathrm{T}}(x, \bar{y}(x), \bar{y}'(x))$.

1.4 Uniform noise

If $\sigma_{\mathrm{T}}(x, \bar{y}(x), \bar{y}'(x)) = \sigma$, i.e. for uniform noise, $\partial\mathcal{L}/\partial y = 0$ and Eq. (S7) becomes:

$$\begin{cases} p = \dfrac{\bar{y}'}{Z\sigma} & \text{(S19)} \\ \dfrac{d}{dx}\left(\dfrac{p}{\bar{y}'}\right) = 0. & \text{(S20)} \end{cases}$$

Eq. (S19) clearly solves Eq. (S20) and $Z = 1/\sigma \int dx\ \bar{y}'(x)$ is a normalization constant. Note that as shown in Sec 1.1 normalizing the input distribution is equivalent to adding a Lagrangian multiplier to the Eq. S7. In other words, the optimization problem is independent from the normalization factor of the input distribution. For simplicity, we drop the Lagrangian multiplier in the following treatment. In this case, the two approaches, namely maximization with respect to the input distribution and maximization with respect to the input-output curve, provide the same result. This means that in order to perform the maximization we need to know either the input distribution or the input-output curve.

The result in Eq. (S19) was first obtained by Laughlin, finding that the probability of output is flat (equivalent to histogram equalization in image analysis) and that the input-output curve is the cumulative integral of the input distribution (i.e. $\bar{y}' \propto p$ see Fig. S1) [12].

1.5 Input noise only

If $\sigma_{\mathrm{T}}(x, \bar{y}(x), \bar{y}'(x)) = \sigma_{\mathrm{x}}(x)$, the solution of Eq. (S7) is again Eqs. (S19)-(S20), with the only difference that $Z = \int dx\ y'(x)/\sigma_{\mathrm{x}}(x)$.

1.6 Output noise only

If $\sigma_{\mathrm{T}}(x,\bar{y}(x),\bar{y}'(x))=\sigma_{\mathrm{y}}(\bar{y})$, the first of Eq. (S7) gives again the result of Eq. (S19), while the second equation

$$-\frac{p}{\sigma_{\mathrm{y}}}\frac{d\sigma}{dy}=\frac{d}{dx}\frac{p}{\bar{y}'} \tag{S21}$$

can be written as

$$-\frac{\sigma_{\mathrm{y}}'}{\sigma_{\mathrm{y}}}-\frac{p'}{p}+\frac{\bar{y}''}{\bar{y}'}=0. \tag{S22}$$

Again, Eq. (S19) solves Eq. (S22) with $Z=\int dx\ y'\bar{(}x)/\sigma_{\mathrm{T}}(\bar{y}(x))$, and the two approaches are equivalent.

1.7 Independent transmitted input and output noise

If $\sigma_{\mathrm{T}}(x,\bar{y}(x),\bar{y}'(x))=\sqrt{\sigma_{\mathrm{x}}^2(x)\bar{y}'^2+\sigma_{\mathrm{y}}^2(\bar{y})}$, the solution of Eq. (S7) is

$$\begin{cases} p=\dfrac{\bar{y}'}{Z\sigma_{\mathrm{T}}} & \text{(S23)}\\ 2\dfrac{\bar{y}''}{\bar{y}'}-2\dfrac{\sigma_{\mathrm{T}}'}{\sigma_{\mathrm{T}}}+\dfrac{\frac{d}{dx}\frac{\partial\sigma_{\mathrm{T}}}{\partial\bar{y}'}}{\frac{\partial\sigma_{\mathrm{T}}}{\partial\bar{y}'}}=-\dfrac{1}{\bar{y}'}\dfrac{\frac{\partial\sigma_{\mathrm{T}}}{\partial x}}{\frac{\partial\sigma_{\mathrm{T}}}{\partial\bar{y}'}}. & \text{(S24)}\end{cases}$$

Note that Eq. (S23) is equivalent to $-\frac{\sigma_{\mathrm{T}}'}{\sigma_{\mathrm{T}}}-\frac{p'}{p}+\frac{\bar{y}''}{\bar{y}'}=0$ once differentiated (see *Materials and Methods* in the main text for derivation). Eq. (S24), when written in terms of σ_{x} and σ_{y}, becomes

$$2\frac{\bar{y}''}{\bar{y}'}-2\frac{\sigma_{\mathrm{T}}'}{\sigma_{\mathrm{T}}}+\frac{\frac{d}{dx}\frac{\partial\sigma_{\mathrm{T}}}{\partial\bar{y}'}}{\frac{\partial\sigma_{\mathrm{T}}}{\partial\bar{y}'}}=-\frac{\frac{\partial\sigma_{\mathrm{x}}^2}{\partial x}}{2\sigma_{\mathrm{x}}^2}, \tag{S25}$$

and after integration over x, we obtain

$$\log\left(\frac{\bar{y}'^3\sigma_{\mathrm{x}}^3}{\sigma_{\mathrm{T}}^3}\right)=K, \tag{S26}$$

where K is an integration constant given by the boundary conditions. Finally, we can rewrite the result as

$$\sigma_{\mathrm{x}}^2\bar{y}'^2=Q\sigma_{\mathrm{y}}^2, \tag{S27}$$

where $Q=1/(1-\mathrm{e}^{2K/3})$. In this case, given the noise we can maximize the mutual information with respect to both the input distribution and the input-output curve. The maximal mutual information, found by using Eq. (S23) in Eq. (S5), is $\mathcal{I}=\log_2\sqrt{2\pi\mathrm{e}}Z$, where $Z=\int \mathrm{d}x\ \bar{y}'/\sigma_T$ depends on the optimal response $\bar{y}$. Note that if the noise increases (decreases) by a multiplying factor the mutual information decreases (increases) in amplitude. However, the optimal input distribution and input-output curve remain the same. In the following we focus on different types of input and output noise.

1.7.1 Noise: $\sigma_{\mathbf{T}}(x,\bar{y}(x),\bar{y}'(x))=\sqrt{\alpha_1 x\bar{y}'^2+\alpha_4}$, with constants $\alpha_{1,4}$

Eq. (S27) becomes

$$\bar{y}'^2=\frac{Q\alpha_4}{\alpha_1 x}, \tag{S28}$$

and the solution for the input-output curve is

$$\bar{y} = \pm 2\sqrt{\frac{Q\alpha_4}{\alpha_1}}\sqrt{x} + C, \tag{S29}$$

where C is an integration constant. We choose C and K, and hence Q, in order to have a response ranging from 0 to 1 in the sensitive region of the receptors, i.e. $\bar{y}(x_{\text{off}}) = 1$ and $\bar{y}(x_{\text{on}}) = 0$. Note that first, the boundary conditions also determine which of the two solution in Eq. (S29) is the correct one. Second, since Q appears with a multiplicative factor α_4/α_1, we expect that the final solution will not depend on those parameters. Indeed, after imposing the boundary conditions, Eq. (S29) becomes

$$\bar{y} = -\frac{\sqrt{x}}{\sqrt{x_{\text{on}}} - \sqrt{x_{\text{off}}}} + \frac{\sqrt{x_{\text{on}}}}{\sqrt{x_{\text{on}}} - \sqrt{x_{\text{off}}}}, \tag{S30}$$

which is independent of α_1 and α_4.

1.7.2 Noise: $\sigma_{\mathbf{T}}(x, \bar{y}(x), \bar{y}'(x)) = \sqrt{\alpha_1 x \bar{y}'^2 + \alpha_3 \bar{y} + \alpha_4}$, with constants $\alpha_{1,3,4}$

Eq. (S27) becomes

$$\alpha_1 x \bar{y}'^2 = Q(\alpha_4 + \alpha_3 \bar{y}) \longrightarrow \frac{\bar{y}'}{\sqrt{\alpha_4 + \alpha_3 \bar{y}}} = \pm\sqrt{\frac{Q}{\alpha_1 x}} \tag{S31}$$

and the solution for the input-output curve is

$$\bar{y} = \frac{Q\alpha_3}{\alpha_1}x \pm \alpha_3 C\sqrt{\frac{Q}{\alpha_1}}\sqrt{x} + \left(\frac{\alpha_3 C^2}{4} - \frac{\alpha_4}{\alpha_3}\right), \tag{S32}$$

where C is an integration constant. We choose C and K, and hence Q, in order to have a response ranging from 0 to 1 in the sensitive region of the receptors, i.e. $\bar{y}(x_{\text{off}}) = 1$ and $\bar{y}(x_{\text{on}}) = 0$. Note that x appears with a prefactor Q/α_1, which is set by boundary conditions and makes the equation for the optimal input-output curve independent of α_1.

1.7.3 Noise: $\sigma_{\mathbf{T}}(x, \bar{y}(x), \bar{y}'(x)) = \sqrt{\alpha_1 x \bar{y}'^2 + \alpha_2 \bar{y}(1-\bar{y}) + \alpha_3 \bar{y} + \alpha_4}$, with constants α_{1-4}

Eq. (S27) becomes

$$\alpha_1 x \bar{y}'^2 = Q\left(\alpha_4 + (\alpha_3 + \alpha_2)\bar{y} - \alpha_2 \bar{y}^2\right) \longrightarrow \frac{\bar{y}'}{\sqrt{\alpha_4 + (\alpha_3 + \alpha_2)\bar{y} - \alpha_2 \bar{y}^2}} = \pm\sqrt{\frac{Q}{\alpha_1 x}} \tag{S33}$$

and the solution for the input-output curve is

$$\bar{y} = \frac{\alpha_2 + \alpha_3 \pm \sqrt{(\alpha_2 + \alpha_3)^2 + 4\alpha_2\alpha_4}\,\sin\left[\sqrt{\alpha_2}\left(C + \frac{2\sqrt{Qx}}{\sqrt{\alpha_1}}\right)\right]}{2\alpha_2}, \tag{S34}$$

where C is an integration constant. We choose C and K, and hence Q, in order to have a response ranging from 0 to 1 in the sensitive region of the receptors, i.e. $\bar{y}(x_{\text{off}}) = 1$ and $\bar{y}(x_{\text{on}}) = 0$. Once again, x appears with a prefactor Q/α_1, which is set by boundary conditions and makes the equation for the optimal input-output curve independent of α_1 (see Fig. S3 for an analysis of the sensitivity of the solution on different choices of parameters α_{2-4}).

1.7.4 Boundary conditions on $\bar{y}'$ that produce smooth input-output curves

Given the noise $\sigma_\mathrm{T}(x,\bar{y}(x),\bar{y}'(x)) = \sqrt{\alpha_1 x \bar{y}'^2 + \alpha_2\bar{y}(1-\bar{y}) + \alpha_3\bar{y} + \alpha_4}$, with constants α_{1-4} as in Sec. 1.4.3, we now explore the possibility to set the boundary conditions in such a way that the first derivative of the input-output curve is zero at the boundaries, i.e. $\bar{y}'(x_\mathrm{off}) = 0$ and $\bar{y}'(x_\mathrm{on}) = 0$. The general solution before imposing any conditions on the boundaries is Eq. (S30), and thus the first derivative is

$$\bar{y}' = \pm\sqrt{\frac{\left((\alpha_2+\alpha_3)^2 + 4\alpha_2\alpha_4\right)Q}{4\alpha_1\alpha_2 x}}\cos\left[\sqrt{\alpha_2}\left(C + \frac{2\sqrt{Qx}}{\sqrt{\alpha_1}}\right)\right]. \tag{S35}$$

To set the boundary conditions $\bar{y}'(x_\mathrm{off}) = 0$ and $\bar{y}'(x_\mathrm{on}) = 0$ without having the function $\bar{y}'$ identically zero for any x, we need to impose that the argument of the cosine is equal $\pi/2 + n\pi$ with $n \in \mathcal{N}$. However, the argument of the cosine is the same as the argument of the sine in Eq. (S34). This means that by setting $\bar{y}'(x_\mathrm{off}) = 0$ and $\bar{y}'(x_\mathrm{on}) = 0$, we obtain $\bar{y}(x_\mathrm{off}) = \frac{\alpha_2+\alpha_3 \pm\sqrt{(\alpha_2+\alpha_3)^2+4\alpha_2\alpha_4}}{2\alpha_2}$ and $\bar{y}(x_\mathrm{on}) = \frac{\alpha_2+\alpha_3 \mp\sqrt{(\alpha_2+\alpha_3)^2+4\alpha_2\alpha_4}}{2\alpha_2}$. However, this way the input-output curve becomes negative when α_4 is different than zero. Since biologically negative concentrations of outputs would be unreal, we reject this solution. However, in the case in which the constant output noise is negligible, i.e. $\alpha_4 \ll \alpha_{2,3}$, the input-output curve, after setting the first derivatives at the boundaries to zero, becomes

$$\bar{y} \approx \frac{1+\alpha_3/\alpha_2}{2}\left(1 \pm \sin\left[\frac{\pi\left(-3 + 2\sqrt{x/x_\mathrm{off}} + \sqrt{x_\mathrm{on}/x_\mathrm{off}}\right)}{2\left|-1+\sqrt{x_\mathrm{on}/x_\mathrm{off}}\right|}\right]\right), \tag{S36}$$

which depends on the ratio α_3/α_2. In particular fulfilling the condition $\bar{y}(x_\mathrm{off}) = 1$ requires $\alpha_3 = 0$ (no switching noise). In other words, the solution with first derivatives equal to zero at the boundaries implies that the signaling noise is the dominant internal source of noise. The final solution is plotted in Fig. S3.

1.7.5 Inverse problem: predicting internal noise for an optimal Hill-function solution

Here, we invert the problem: given a Hill function as the optimal solution, i.e. $\bar{y}(x) = k^n/(x^n + k^n)$, we extract the noise terms. Assuming independent transmitted input and output noise, we obtain Eq. (S27). We further assume an input noise proportional to the strength of the input ($\sigma_x^2 = \alpha_1 x$), i.e. Poisson noise [13], and focus on the output noise σ_y. Hence, Eq. (S27) becomes

$$Q\sigma_y^2 = \alpha_1 x\left(-\frac{k^n n x^{n-1}}{(k^n+x^n)^2}\right)^2. \tag{S37}$$

Thus, the output noise as a function of the input x is

$$\sigma_y^2 = \frac{\alpha_1}{Q}\frac{k^{2n}n^2x^{2n-1}}{(k^n+x^n)^4}. \tag{S38}$$

By inverting the Hill function, $x = k^n\left(\frac{1-\bar{y}}{y}\right)^{\frac{1}{n}}$, the output noise can be written as function of output $\bar{y}$,

$$\sigma_y^2 = \frac{\alpha_1 n^2\bar{y}^4}{Qk^{2n}}\left(k^n\frac{1-\bar{y}}{\bar{y}}\right)^{\frac{2n-1}{n}}. \tag{S39}$$

Note that in the main text we state that the solution for the maximization of the mutual information with respect to both the input distribution and the input-output curves implies that the optimal transmitted input noise and output noise are proportional (Eq. (S27)). Hence, the total noise becomes

$$\sigma_T^2 = (Q+1)\sigma_y^2, \tag{S40}$$

which is a function of the optimal input-output curve $\bar{y}$. In Fig. S4, we compare the total standard deviation σ_T for the different types of output noise presented in this section, showing that σ_T^2 has an unimodal peaked structure.

1.7.6 The optimal input distribution only depends on the input noise

Going back to the system of Eqs. (S23)-(S24) for independent transmitted input and output noise, we showed that the solution of the joint optimization is

$$\begin{cases} p = \dfrac{\bar{y}'}{Z\sigma_{\mathrm{T}}} \\ \sigma_{\mathrm{x}}^2 \bar{y}'^2 = Q\sigma_{\mathrm{y}}^2. \end{cases}$$

Substituting the second equation into the first, we notice that

$$p = \frac{\bar{y}'}{Z\sqrt{\left(1+\frac{1}{Q}\right)\sigma_{\mathrm{x}}^2 \bar{y}'^2}} = \frac{1}{Z\sqrt{\left(1+\frac{1}{Q}\right)}\sigma_{\mathrm{x}}}. \tag{S41}$$

This equation suggest that for the optimal input-output curve the input distribution depends only on the input noise. In our case, the input noise is normally set by the Berg-and-Purcell limit, i.e. $\sigma_{\mathrm{x}} = \sqrt{\alpha_1 x}$. Thus, Eq. (S41) becomes

$$p(x) = \left[2\left(\sqrt{x_{\mathrm{on}}} - \sqrt{x_{\mathrm{off}}}\right)\sqrt{x}\right]^{-1},$$

which is plotted in Fig. S3D. This shows that for Berg-and-Purcell input noise, the input distribution is universal, i.e. independent of the output noise, while the input-output curve is not universal.

1.8 Discussion of the sensitive region

The effect of changing the size of the sensitive region for the individual maximizations with respect to the input distribution and the input-output curve is shown in Fig. S2. Given (or targeting) a Hill function with a certain Hill coefficient, the mutual information increases when enlarging the range of sensitivity and eventually levels off due to the saturation of the response.

For the joint maximization approach the sensitive region plays an important role in setting the steepness of the response: the larger the sensitive regime, the smoother the response and the broader the input distribution that maximizes the joint optimization. However, also in this case, the mutual information saturates for increasing the sensitive region, since the positive effect of integrating the information over a broader region is counterbalanced by a flattering, uninformative input-output curve (Fig. S2). Tkacik and co-workers in [10] explored numerically those properties for the case of Hill functions of variable Hill coefficient. In our manuscript, we take a different approach; we assume the sensitive region is fixed by the nature of the receptors and we then optimize analytically and numerically the mutual information with respect to both the input distribution and the input-output curve.

As explained in details in Secs. 3 and 4.3, the sensitive regions for the receptor channel for the different methylation levels are taken from data [1]. Those values also set the sensitive region of the motor channel $y \in [\bar{y}(k_{\mathrm{on}}), \bar{y}(k_{\mathrm{off}})]$ and are expressed in units of the total concentration of CheY $[Y_T]$. Note that the total CheY concentration can never be fully phosphorylated [14].

2 Mutual information and Fisher information

A key problem in Bayesian statistics is estimating the value of a parameter of an underling probability distribution from a set of observable outcomes of a random process. For instance, we want to estimate

the parameter x from a set of outcomes of a random variable, $\boldsymbol{y} = \{y_1, ..., y_n\}$, having a model $p(\boldsymbol{y}|x)$, which in Bayesian statistics is called likelihood. The initial probability distribution of observing a certain value of the parameter is called the prior distribution, $p(x)$, since it reflects an assumption before taking into account any evidence, i.e. the outcomes. After observing the outcomes we can evaluate the posterior distribution $p(x|\boldsymbol{y}) \propto p(\boldsymbol{y}|x)p(x)$.

Of great interest for the theory is to identify the priors which do not influence the posterior distribution, called 'uninformative priors'. For instance, one can naively assume that without knowing anything about the parameter the possible values it can assume are uniformly distributed (uniform prior). However, under a different parametrization this prior may not be uniform, making the problem of setting a prior nontrivial. A way of formalizing the uninformative priors was introduced by Bernardo in terms of 'reference priors' [15], which are an extension of Jeffreys priors [16].

The reference priors are the priors which maximize the Kullback-Leibler divergence between the posterior and the prior distribution

$$\mathrm{KL}[p(x), p(x|y)] = \int \mathrm{d}x \; p(x|y) \log \frac{p(x|y)}{p(x)}. \tag{S42}$$

The expectation value over all possible outcomes is the mutual information,

$$\mathcal{I}[x, y] = \int \mathrm{d}y \; p(y) \int \mathrm{d}x \; p(x|y) \log \frac{p(x|y)}{p(x)}. \tag{S43}$$

This means that to find the reference prior we need to find the maximal mutual information with respect to $p(x)$, i.e. the channel capacity. Note that here we restrict our analysis to $\boldsymbol{y} = \{y\}$, but the reference priors provide a framework for generalizing to higher dimensions.

The Fisher information of an unknown parameter x, extracted from observing the outcomes of a random variable $\boldsymbol{y} = \{y_1, ..., y_n\}$, is mathematically defined by

$$\mathcal{F}(x) = -\int \mathrm{d}^n y \, p(\boldsymbol{y}|x) \left(\frac{\partial^2}{\partial x^2} \log p(\boldsymbol{y}|x) \right). \tag{S44}$$

In general, it is possible to extract x from the outcomes using an estimator $\hat{x} = \hat{x}(\boldsymbol{y})$. An estimator is unbiased if the expected value of the estimator is equal to the true value of the parameter being estimated, i.e. $\langle \hat{x} \rangle = \int \mathrm{d}^n y \; p(\boldsymbol{y}|x)\hat{x}(\boldsymbol{y}) = x$. The variance of such an unbiased estimator is linked to the Fisher information via the Cramér-Rao bound

$$\sigma_x^2 = \left\langle (\hat{x}(\boldsymbol{y}) - x)^2 \right\rangle_x \geq \frac{1}{\mathcal{F}(x)}, \tag{S45}$$

where now the Fisher information is calculated using $p(\hat{x}|x)$ instead of $p(\boldsymbol{y}|x)$. If the equality holds the estimator is said to be 'efficient'. Note that the maximum-likelihood estimation is asymptotically efficient (for large n). In other words, the Fisher information provides a lower bound on the estimate of an unbiased parameter x.

To connect the Fisher to the mutual information, we follow Brunel and Nadal [7]. We consider the mutual information between an input X and an output Y:

$$\mathcal{I}[Y, X] = \int \mathrm{d}y \, \mathrm{d}x \, p(x) p(y|x) \log \frac{p(y|x)}{p(y)}. \tag{S46}$$

If there is an unbiased estimator $\hat{x} = \hat{x}(y)$ with mean x and variance $1/\mathcal{F}(x)$, the mutual information

between x and $\hat{x}$ is

$$\begin{aligned}\mathcal{I}[x,\hat{x}] &= -\int \mathrm{d}\hat{x}\, p(\hat{x}) \log p(\hat{x}) + \int \mathrm{d}x\, p(x) \int \mathrm{d}\hat{x}\, p(\hat{x}|x) \log p(\hat{x}|x) \\ &= -\int \mathrm{d}\hat{x}\, p(\hat{x}) \log p(\hat{x}) - \int \mathrm{d}x\, p(x) \log \sqrt{2\pi \mathrm{e} \sigma_x^2} \\ &\geq -\int \mathrm{d}\hat{x}\, p(\hat{x}) \log p(\hat{x}) - \int \mathrm{d}x\, p(x) \log \sqrt{\frac{2\pi\mathrm{e}}{\mathcal{F}(x)}}, \end{aligned} \tag{S47}$$

where Eq. (S47) uses the fact that the entropy of a Gaussian distribution of variance $1/\mathcal{F}(x)$ is larger or equal than the entropy of any other distribution with the same variance [7, 17]. In the limit of small noise $\mathcal{F}(x) \gg 1$, the entropy of the estimator is equal to the entropy of the variable, resulting in $\int \mathrm{d}\hat{x}\, p(\hat{x}) \log p(\hat{x}) = \int \mathrm{d}x\, p(x) \log p(x)$. Finally, since information can be only lost during processing, we have

$$\mathcal{I}[y,x] \geq -\int \mathrm{d}x\, p(x) \log p(x) - \int \mathrm{d}x\, p(x) \log \sqrt{\frac{2\pi\mathrm{e}}{\mathcal{F}(x)}} = -\int \mathrm{d}x\, p(x) \log \sqrt{\frac{2\pi\mathrm{e}}{\mathcal{F}(x)}} p(x). \tag{S48}$$

In the main text, we show the case of a single outcome y. However, Eq. (S48) can easily be generalized to n outcomes $\boldsymbol{y} = \{y_1, ..., y_n\}$ using

$$\mathcal{I}[\boldsymbol{y},x] \geq -\int \mathrm{d}x\, p(x) \log \sqrt{\frac{2\pi\mathrm{e}}{\mathcal{F}(x)}} p(x), \tag{S49}$$

where $\mathcal{F}(x)$ is calculated from $p(\boldsymbol{y}|x)$. Hence, the mutual and the Fisher information are connected by inequality (S48). However, when the estimator has a Gaussian distribution, or more generally when an efficient estimator exists, equality in Eq. (S49) is expected to hold. This is normally the case when the number of outcomes is large and maximum-likelihood estimation can be used [7]. When equality holds, maximizing the mutual information in Eq. (S48) with respect to distribution $p(x)$, i.e. the channel capacity, leads to

$$p(x) \propto \sqrt{\mathcal{F}(x)}. \tag{S50}$$

Finally, for biased estimators $\hat{x} = \hat{x}(y)$, where $\langle \hat{x} \rangle = \int \mathrm{d}y\; p(y|x)\hat{x}(y) = f(x) \neq x$, the Cramér-Rao bound is

$$\sigma_x^2 \geq \frac{\left(\frac{\mathrm{d}f}{\mathrm{d}x}\right)^2}{\mathcal{F}(x)}. \tag{S51}$$

Generalizing to biased estimators, i.e. using Eq. (S51) instead of Eq. (S45) in Eq. (S47), Eq. (S48) becomes Eq. (S5) (Eq. (2) in the main text). The interested reader can find additional details and the general case of non-Gaussian channels in [7].

3 Sources of noise and estimation of noise parameters

To evaluate the noise parameters α's and β's we use results from [18]. The theory in [18] starts from the dynamical equations governing the receptor activity and then evaluates the variance of the activity of the total cell A_{tot} from the integrated noise power spectra of the activity [18]. This produces the following contributions to the variance from ligand concentration (c), signaling (s) and switching (w) noise:

$$\left\langle \delta A_{\mathrm{tot}}^2 \right\rangle_c \approx \frac{N_{tot}}{N D s \tau} \left(c \frac{\partial A}{\partial c} \right)^2 \tag{S52a}$$

$$\left\langle \delta A_{\text{tot}}^2 \right\rangle_m \approx \frac{2N_{tot}\gamma_R A(1-A)}{\omega_m} \tag{S52b}$$

$$\left\langle \delta A_{\text{tot}}^2 \right\rangle_w \approx \frac{2k_2 N_{tot} A}{\pi\tau(k_1+k_2)^2}, \tag{S52c}$$

where N_{tot} is the total number of receptors in the cell, N is the number of receptor in each receptor cluster, τ is the time over which the receptor integrate the binding and unbinding events, s is the size of each receptor cluster, D is the diffusion coefficient, ω_m is the characteristic frequency coming from the adaptation dynamics, γ_R is the rate of receptor methylation, and k_1 and k_2 are the rates of switching from inactive to active and vice versa, respectively. Although these equations were originally derived within the small-noise approximation, we assume their general applicability away from the steady state.

Note that the receptor-cluster activity A in those equations is normalized so that it is either 0 (fully inactive) or 1 (fully active). This means that the total cell activity A_{tot} ranges from 0 (when all clusters are inactive) to maximally N_{tot}/N, i.e. the number of clusters (when all clusters are active). Hence, to get a normalized total cell activity, the noise terms need to be divided by $(N_{tot}/N)^2$. In addition, in the main text we consider Y_p rather than the total activity as output of the receptor channel. However, in a quasi-steady state assumption, we can assume for simplicity that $A = Y_p/Y_{tot}$ with Y_{tot} the total CheY concentration. In this way we have an estimation for the parameters appearing in the main text

$$\alpha_1 \approx \frac{N}{N_{tot} D s \tau Y_{tot}} \sim 10^{-4} \tag{S53a}$$

$$\alpha_2 \approx \frac{2N^2\gamma_R}{N_{tot}\omega_m Y_{tot}} \sim 10^{-4} \tag{S53b}$$

$$\alpha_3 \approx \frac{2k_2 N^2}{\pi N_{tot}\tau(k_1+k_2)^2 Y_{tot}^2} \sim 10^{-4}, \tag{S53c}$$

with the exception of $\alpha_4 \approx 10^{-5}$, which represents an arbitrary background noise to regulate the numerical evaluation of the mutual information. Note that we always assume $\alpha_4 \ll \alpha_{1,2,3}$. Importantly Eqs. (S53a)-(S53c) contain dynamic rate constants for noise filtering. The parameter values are provided in Tab. S1. The β parameters of the motor noise have a similar meaning as the α parameters at the receptors. FliM can be in CW or CCW states, and the motor adapts by changing the number of constituent molecules [19–22]. For this reason we assume them to be of the same order of magnitude as the α parameters ($\beta_{2,3,4} \sim 10^{-4} - 10^{-5}$). In Sec. 4.2, we explore the robustness of our results to changes in α and β values.

4 Additional results

4.1 Optimal information transmission for uniform noise

Fig. S1 shows the Laughlin approach for maximizing to mutual information for uniform noise [12]. Given the input distribution we can find the input-output curve and vice versa.

4.2 Noise sensitivity

4.2.1 Joint optimization for different α values

Fig. S3 shows the sensitivity of the optimal input-output curve and optimal input distribution to changes in the noise parameters α_{1-4}. As discussed in Sec. 1.4, the input-output curve is independent of changes in α_1, i.e. the amplitude of the input noise, while the optimal input distribution is universal given the input noise.

4.2.2 Total noise for optimal information transmission for different types of output noise

The total noise of a single channel which underwent joint optimization is shown in Fig. S4. In particular, the total noise in Eq. (S40) is plotted for the different types of output noise presented in Sec. 1.7.1 - 1.7.4, i.e uniform background output noise ($\alpha_4 \neq 0$, $\alpha_{2,3} = 0$), uniform background output noise and signaling noise ($\alpha_{3,4} \neq 0$, $\alpha_2 = 0$), uniform background output noise, signaling noise and switching noise ($\alpha_{2,3,4} \neq 0$). Finally, we also show the shape of the total noise as a function of the input for the case of the Hill function as the optimal input-output curve, as presented in Eq. (S40). Note that the values of the α parameters have been tuned to reach an approximately equivalent total noise level (defined as the integral of the noise over the input).

4.2.3 Comparison of the total noise at the receptor and motor channels

Here, we compare the total noise of the receptor and motor channels. Briefly the channels are now defined by the Hill coefficients n and m for the receptor and motor, respectively. The two channels are considered independently, in agreement with the assumptions described in the Result section entitled "Information transmission along the *E. coli* chemotaxis pathway" in the main text. The noise of the receptor channel is $\sigma_{yT}(x, y, G_y) = \sqrt{\alpha_1 x\ G_Y^2 + \alpha_2 \bar{y}(1-\bar{y}) + \alpha_3 \bar{y} + \alpha_4}$, which can be written as a function of $\bar{y}$ by inverting the relationship $\bar{y}(A(c)) = \frac{k_Y A(c)}{k_Y A(c) + k_Z Z + \gamma_Y}$ with $A(c)$ the Hill equation of the receptor activity as a function of the external concentration, and k_Y, k_Z, γ_Z and Z are constants (see [14] for details). The noise for the motor channel is $\sigma_{zT}(y, z, G_z) = \sqrt{\sigma_{zT}^2 G_z^2 + \beta_2 \bar{z}(1-\bar{z}) + \beta_3 \bar{z} + \beta_4}$, which is a function of y when $\bar{z}(y)$ is the Hill equation of the motor channel.

Fig. S5 show the shape of σ_{yT} and σ_{zT} as a function of y for a particular value of n and m (panel A). The plot explores the ratio σ_{yT}/σ_{zT} as a function of n and y (Fig. S5B), m and y (Fig. S5C), and the ratio of the integrated noise over y as a function of m and n (Fig. S5D). In general, the noise at the motor is larger than the noise at the receptor.

4.2.4 High information transmission for multiple motors with high Hill coefficients is robust to changes in noise level

Fig. S6 reproduces the result of Fig. 5 in the main text for different values of the β parameters. In particular, the high information transmission for multiple motors at high Hill coefficients is maintained even when setting the switching noise, the signaling noise, or the uniform background noise to zero (not simultaneously).

4.3 Comparison of optimal input-output curves with experimental data from [1]

Here, we compare the optimal input-output curves from our joint optimization with the established Monod-Wyman-Changeux (MWC) model for signaling by cooperative receptor clusters and fluorescence resonance energy transfer (FRET) data in *E. coli* [1].

In the MWC model the receptor cluster can either be in the active (*on*) or inactive (*off*) state, and the resulting activity of a single cluster is given by

$$A = \frac{1}{1 + \mathrm{e}^F}, \tag{S54}$$

where the free-energy difference F depends on the concentration of extracellular ligand c and methylation level m

$$F(c, m) = N\left[\epsilon(m) + \sum_r \nu_r \log\left(\frac{1 + c/K_r^{\mathrm{off}}}{1 + c/K_r^{\mathrm{on}}}\right)\right], \tag{S55}$$

where N and $\epsilon(m)$ are the number of receptors in a cooperative receptor cluster and the energy at given methylation level, respectively. K_r^{on} and K_r^{off} are the ligand dissociation constants in the *on* and *off* state, respectively, and r denotes the type of receptor with fraction ν_r in the cluster. Here, we consider only Tar receptors.

Wild-type *E. coli* cells have 4 methylations sites for each receptor and the proteins CheR and CheB determine the methylation level by constantly methylating and de-methylating the receptor sites, respectively. To measure the input-output curves experimentally, cheRcheB mutants, unable to express CheR and CheB, are used [23]. The methylation sites are genetically modified in order to have either the amino acids glutamate (E) for unmethylated sites or glutamine (Q) to mimic methylated sites. Hence, by setting the number of Q's fixes the methylation level, e.g. mutant EEEE corresponds to unmethylation receptors, mutant QEEE has methylation level 2 (one Q for each receptor in a dimer), QEQE has methylation level 4, QEQQ has 6, and finally QQQQ has 8 [23].

To extract the sensitive region (x_{on}, x_{off}) at fixed modification level, we fixed the modification level m (number of Q's) and used N and $\epsilon(m)$ from [1]. The sensitivity is defined by $\delta A/(\delta c/c)$ [24]. Thus, the sensitive regime is found by solving

$$\frac{\delta A}{\delta c/c} = \frac{\max\{\frac{\delta A}{\delta c/c}\}}{2}\Big|_{\mathrm{m=m^*}} \tag{S56}$$

for c, where m^* corresponds to the fixed modification level of the mutants.

In order to perform our joint optimization a few assumptions are required. Firstly, we made educated guesses about the functional form of the noise, and the noise parameters α_{1-4}. However, our results are not sensitive to the exact α values, and in the following they are chosen in order to maintain the observed average noise level (see Fig. S4). In addition to setting the noise terms, the joint optimization requires specifying the sensitive regime of the receptors, which we extracted from FRET data as described next (alternatively they can be determined from biochemical estimates of the ligand dissociation constants [25–27]).

After guessing the noise and extracting the the sensitive regime of receptor, we calculated Eq. (S34). Fig. S9A compares our solution with the MWC model in [1]. To quantify this comparison, we fitted both models to Hill functions, and we compare the Hill coefficients in Fig. S9B. Overall, the comparison is good for QEQQ and QQQQ receptors. Finally, by calculating $\mathcal{I}^{\max} = \log \frac{Z}{\sqrt{2\pi \mathrm{e}}}$, where $Z = \int_{x_{\min}}^{x_{\max}} \mathrm{d}x \, \frac{\bar{y}'(x)}{\sigma_{\mathrm{T}}(x)}$, for both models, we showed that the level of mutual information achieved using our joined optimization is considerably larger than the one reached with the response curves from the MWC model (see Fig. S9B).

In order to compare with FRET data, note that FRET measures $\mathrm{CheY_p} - \mathrm{CheZ}$ pairs, which reflect the $\mathrm{CheY_p}$ level under the assumption of constant and uniform phosphatase concentration CheZ [28]. The activity of a receptor A is related to $\mathrm{CheY_p}$ at quasi-steady state via $[\mathrm{CheY_p}] = \frac{k_Y A[\mathrm{CheY^T}]}{k_Y A + k_Z[\mathrm{CheZ}] + \gamma_Y}$, where k_Y, k_Z and γ_Y are constants [14]. This assumption is justified since phosphorylation of CheY through $\mathrm{CheA_p}$ occurs fast (order of 10 ms). Under these assumptions $\mathrm{CheY_p}$ is a readout of the receptor activity [14, 28, 29].

4.4 Mutual information between $\mathrm{CheY_p}$ and motor bias for multiple motors

To calculate the mutual information between a single input y, representing the $\mathrm{CheY_p}$ concentration, and multiple outputs $z_1, ..., z_k$, representing the clockwise biases of K motors, we use the chain rule for the mutual information

$$\mathcal{I}[y; z_1, ..., z_K] = \sum_{i=1}^{K} \mathcal{I}[z_i; y|z_{i-1}, ..., z_1], \tag{S57}$$

with the conditional mutual information $\mathcal{I}[X;Y|Z] := \int dx\ dy\ dz\ p(x,y,z)\ \log \frac{p(x,y|z)}{p(x|z)\ p(y|z)}$ [30]. As an example, we start with two motors. Thus, Eq. (S57) becomes $\mathcal{I}[y;z_1,z_2] = \mathcal{I}[z_1;y] + \mathcal{I}[z_2;y|z_1]$. If the motors are coupled, $p(z_1,z_2) = p(z_1)$, then $\mathcal{I}[y;z_1,z_2] = \mathcal{I}[y;z_1]$ and the information does not increase beyond the single motor, independent of the number of motors. However, in the most general case the mutual information has no upper limit due to potential negative correlations, which can lead to cancellation of noise [31]. However, in our case we assumed (and verified with simulations in Sec. 4.6) that the motors are conditionally independent, i.e. independent given the $\mathrm{CheY_p}$ concentration $p(z_1,z_2,y) = p(z_1|y)p(z_2|y)p(y)$, leading to

$$\begin{aligned}
\mathcal{I}[y;z_1,z_2] &= \mathcal{I}[z_1;y] + \mathcal{I}[z_2;y|z_1] \\
&= \mathcal{I}[z_1;y] + \int \mathrm{d}y\mathrm{d}z_1\mathrm{d}z_2\ p(z_1,z_2,y)\log_2 \frac{p(z_2,y|z_1)}{p(z_2|z_1)p(y|z_1)} \\
&= \mathcal{I}[z_1;y] + \int \mathrm{d}y\mathrm{d}z_1\mathrm{d}z_2\ p(z_1,z_2,y)\log_2 \frac{p(z_1,z_2,y)}{p(z_2|z_1)p(y|z_1)p(z_1)} \\
&= \mathcal{I}[z_1;y] + \int \mathrm{d}y\mathrm{d}z_1\mathrm{d}z_2\ p(z_1,z_2,y)\log_2 \frac{p(z_2|y)p(z_1|y)p(y)}{p(z_2|z_1)p(y|z_1)p(z_1)} \\
&= \mathcal{I}[z_1;y] + \int \mathrm{d}y\mathrm{d}z_1\mathrm{d}z_2\ p(z_1,z_2,y)\log_2 \frac{p(z_2|y)}{p(z_2|z_1)} \qquad \text{(S58a)} \\
&= \mathcal{I}[z_1;y] - H(z_2|y) + H(z_2|z_1) \qquad \text{(S58b)} \\
&= \mathcal{I}[z_1;y] - \int \mathrm{d}y\ p(y)\log_2\left(\sqrt{2\pi\mathrm{e}}\ \sigma_{zT}(y)\right), \qquad \text{(S58c)}
\end{aligned}$$

where in Eq. (S58c) we note that $H(z_2|y)$ is the Gaussian channel entropy. In the small-noise approximation, we obtain

$$\begin{aligned}
H(z_2|z_1) &= -\int \mathrm{d}y\ \mathrm{d}z_1\ \mathrm{d}z_2\ p(z_1|y)p(z_2|y)p(y)\log_2 p(z_1|z_2) \\
&\approx \int \mathrm{d}y\ \mathrm{d}z_1\ \mathrm{d}z_2\ \delta(z_1-\bar{t}(y))\delta(z_2-\bar{t}(y))p(y)\log_2 p(z_1|z_2) \\
&= -\int \mathrm{d}y\ p(y)\log_2 p\left(\bar{t}(y)|\bar{t}(y)\right) \approx 0\ .
\end{aligned}$$

Eq. (S58b) is easily generalizable to K identical motors

$$\mathcal{I}[y;z_1,..,z_K] = \mathcal{I}[z_1;y] - KH(z_1|y) + \sum_{i=2}^{K} H(z_i|z_{i-1},...,z_1)\ \approx \mathcal{I}[z_1;y] - KH(z_1|y). \qquad \text{(S59)}$$

Making the Gaussian assumption, the conditional entropy $H(z_1|y) = \int \mathrm{d}y\ p(y)\log_2\left(\sqrt{2\pi\mathrm{e}}\sigma_T(y)\right)$ can be calculated numerically. The numerical evaluation of the integrals have been compute using "NIntegrate" function in MATHEMATICA 10. The results are shown in Fig. 5C of the main text and Fig. S6. Finally, note that if in addition to conditional independence, the motor are also independent, i.e. $p(z_1,z_2) = p(z_1)p(z_2)$, Eq. (S58a) becomes $\mathcal{I}[y;z_1,z_2] = 2\mathcal{I}[y;z_1]$, which can be further extended for K motors to $\mathcal{I}[y;z_1,...,z_K] = K\mathcal{I}[y;z_1]$.

4.4.1 Maximization of information transmission for the two-motor channel

Figure 5 shows the optimal information transmission at the motors, and Fig. S6 shows the robustness of the mutual information of the motor channel to changes in β values. The solid and dashed lines in Fig. 5 and Fig. S6 are obtained by maximizing the mutual information for a single-motor channel (solid line), or by obtaining the optimal input distribution for a single motor and calculating the mutual information for two motors using Eq. (S59). Here, we note that Eq. (S59) can be maximized as a function of the input distribution of the two-motor channel by changing the input distribution for the two-motor case. Eq. (S59) can be written as

$$\begin{aligned}\mathcal{I}[y; z_1, .., z_K] &= -\int \mathrm{d}y\ p(y) \log_2 \left[\sqrt{2\pi \mathrm{e}}\ \sigma_{zT}(y)\right] - \int \mathrm{d}y\ p(y) \log_2 \left[\frac{p(y)}{G_z}\right] + \\ &\quad - K \int \mathrm{d}y\ p(y) \log_2 \left[\sqrt{2\pi \mathrm{e}} \sigma_{zT}(y)\right] \\ &= -\int \mathrm{d}y\ p(y) \log_2 \left[\left(\sqrt{2\pi \mathrm{e}}\ \sigma_{zT}(y)\right)^K \frac{p(y)}{G_z}\right]. \end{aligned} \tag{S60}$$

Eq. (S60) can be maximized with respect to $p(y)$ and reaches its optimum for $p(y) = \frac{G_z}{Z(\sigma_{zT}(y))^K}$, with normalization factor Z. The resulting information transmission is shown by dotted lines in Fig. 5 and Fig. S6 for $K = 2$.

4.5 Estimating the mutual information between the external ligand concentration and motor bias

In the previous sections we focused on optimizing the mutual information for a single channel. Indeed, even when the receptor and motor channels are considered to transmit information from the external concentration x to the motor bias z, the two channels were considered separately. Here, we aim to extend the result to the mutual information between x and z, considered as a single channel, i.e. we provide estimations for $\mathcal{I}[x; t] = \int \mathrm{d}x\ \mathrm{d}t\ p(t|x)\ p(x) \log_2 \left(\frac{p(t|x)}{p(t)}\right)$. Using the same approach as in Sec. 1,

$$\mathcal{I}[x; z] = -\int \mathrm{d}x\ p(x) \log_2 \left(\frac{\sqrt{2\pi \mathrm{e}}\ \sigma_{zT}\ p(x)}{G_{zT}}\right), \tag{S61}$$

where G_{zT} is the first derivative of the input-output relationship between x and z. Note that this equation is valid in the small-noise approximation. In addition, the total noise has two contributions. The noise from the receptors, σ_{yT}, is again transmitted to the motor. Furthermore, there is internal noise, σ_z, so that the total noise becomes $\sigma_{zT}^2 = \sigma_{yT}^2 G_z^2 + \sigma_z^2$. Thus, Eq. (S61) has two limiting cases: when the motor noise is much larger than the receptor noise, the solution is equivalent to maximizing the motor channel. In contrast, when the receptor noise is much larger than the motor noise, the solution is equivalent to maximizing the receptor channel.

4.5.1 Mutual information when the motor noise is much smaller than the receptor noise

Here, we focus on the case $\sigma_z^2 \ll \sigma_{yT}^2 G_z^2$. In this case, Eq. S61 simplifies to

$$\mathcal{I}[x; z] = -\int \mathrm{d}x\ p(x) \log_2 \left(\frac{\sqrt{2\pi \mathrm{e}}\ \sigma_{yT}\ G_z\ p(x)}{G_{zT}}\right). \tag{S62}$$

Considering $p(x) = G_y/(Z\sigma_{yT})$, i.e. the input distribution that maximizes the receptor channel, and observing that $G_{zT} = G_z G_y$, Eq. (S62) becomes

$$\mathcal{I}[x;z] = -\int \mathrm{d}x\; p(x) \log_2 \left(\frac{\sqrt{2\pi\mathrm{e}}\; \sigma_{yT}\; G_z G_y}{G_z G_y Z \sigma_{yT}} \right) = \log_2 \left(\frac{Z}{\sqrt{2\pi\mathrm{e}}} \right), \tag{S63}$$

which is the optimal information transmission for the receptor channel.

4.5.2 Mutual information when the receptor noise is much smaller than the motor noise

Here, we focus on the case $\sigma_{yT}^2 G_z^2 \ll \sigma_z^2$. In this case, Eq. S61 simplifies to

$$\mathcal{I}[x;z] = -\int \mathrm{d}x\; p(x) \log_2 \left(\frac{\sqrt{2\pi\mathrm{e}}\; \sigma_z\; p(x)}{G_{zT}} \right). \tag{S64}$$

We consider $p(y) = G_z/(Z'\sigma_{zT})$, i.e. the input distribution that maximizes the motor channel. In addition, observing $p(x) = p(y)G_y$, valid for small noise at the receptors, and $G_{zT} = G_z G_y$, Eq. (S64) becomes

$$\mathcal{I}[x;z] = -\int \mathrm{d}x\; p(x) \log_2 \left(\frac{\sqrt{2\pi\mathrm{e}}\; \sigma_z\; G_y G_z}{G_z G_y Z' \sigma_z} \right) = \log_2 \left(\frac{Z'}{\sqrt{2\pi\mathrm{e}}} \right), \tag{S65}$$

which is the optimal information transmission for the motor channel.

4.5.3 Numerical approach

In the previous sections we focus on the limits in which either the receptor transmitted noise $\sigma_{yT}^2 G_z^2$ or the motor internal noise σ_z^2 is dominating. Here we relax those assumptions. Note that by removing the assumption of small noise, we cannot use the simplified expression of the mutual information in Eq. S5, and hence we cannot write the mutual information as function of the input distribution only (cf. Eq. S4 and S5). For this reason, we assume that both at the receptors and at the motors there is small Gaussian noise of the same order of magnitude, $\sigma_{yT}^2 G_z^2 \sim \sigma_z^2$. We estimate $\mathcal{I}[x;z]$ in two different ways. First, we assume maximal information transmission at the receptors and we use the optimal input distribution $p^r(x)$ to estimate $p^r(y)$. Subsequently, we use $p^r(y)$ to calculate $\mathcal{I}[y;z]$. The mutual information $\mathcal{I}[x;z] = \min\{\mathcal{I}[y;z];\mathcal{I}[x;y]\}$. Second, we assume maximal mutual information at the motors and use the optimal input distribution $p^m(y)$ to estimate $p^m(x)$. Subsequently, we use $p^m(x)$ to calculate $\mathcal{I}[x;y]$. Again, $\mathcal{I}[x;z] = \min\{\mathcal{I}[y;z];\mathcal{I}[x;y]\}$.

Estimation of $\mathcal{I}[x;z]$ based on maximization at the receptors. Here, we consider $p^r(x) = \frac{G_y}{Z\sigma_{yT}}$. In the small-noise approximation, the resulting output distribution of the receptor channel is $p^r(y) = \frac{1}{Z\sigma_{yT}}$. $p^r(y)$ is then consider the input distribution for the motor channel and the mutual information of the motor is $\mathcal{I}^r[y;z] = -\int \mathrm{d}y\; p_{\mathrm{rec}}(y) \log_2 \left[\frac{\sqrt{2\pi\mathrm{e}}\; \sigma_{zT}\; p_{\mathrm{rec}}(y)}{G_z(y)} \right]$. The mutual information can be estimated numerically using ‘NIntegrate’ function in MATHEMATICA 10. The mutual information $\mathcal{I}^r[y;z]$ is plotted in Fig. S7 for a single motor (blue line) and for two motors (green line). Overall, the optimization at the receptors decreases the information transmission at the motors, but the principle remain intact that high mutual information is achieved by multiple conditionally independent motors with high Hill coefficient.

Estimation of $\mathcal{I}[x; z]$ based on maximization at the motors. Here, we consider $p^m(y) = \frac{G_z}{Z\sigma_{zT}}$. In the small-noise approximation, the resulting input distribution of the receptor channel is $p^m(x) = \frac{G_z G_y}{Z\sigma_{zT}}$. The mutual information of the receptor is $\mathcal{I}^m[x; y] = -\int \mathrm{d}x \; p_{\mathrm{mot}}(x) \log_2 \left[\frac{\sqrt{2\pi e} \; \sigma_{yT} \; p_{\mathrm{mot}}(x)}{G_y(x)} \right]$. The mutual information can again be estimated numerically.

4.5.4 The effect of the motor threshold k_d^m on information transmission

Motivated by the fact that the adapted $\mathrm{CheY_p}$ is below the measured threshold k_d^m of the motor response we vary the threshold constant k_d^m of the motor. Specifically, we calculate the maximal mutual information of the motor by varying the motor channel threshold k_d^m (analogous approach used in section 'Estimation of $\mathcal{I}[x; z]$ based on maximization at the motors'). Fig. S8 shows that both for a single (blue) and for two motors (green line) the mutual information peaks at a k_d^m value higher than the adapted $\mathrm{CheY_p}$ concentration (dashed black line). Overall, these results suggest that the experimental motor threshold value is maximizing the information transmission.

4.6 Test of conditional independent motors

The results presented in Sec. 3.6 and Fig. 5C of the main text depend on the assumption of conditionally independent motors. A way to test this assumption is to simulate the cooperative binding of CheY_p molecules to the FliM-ring of the motors, and to compare the probability of binding $p(N_b)$ with N_b the number of bound molecules for one and two motors. If the motors are conditionally independent, we expect to see the same probability for one and two motors.

In the simulations, the CheY_p level ranges from 1 to 4.5 μM (around the measured steady state [14,32]), converted to molecule number (N_{Y_p}). We further assumed that the motors have $N_{tot} = 20$ binding sites (in line with the observed Hill coefficient although real motors may have up to 30 binding sites) and each motor can be in two different states, either rotating clockwise (CW) or counterclockwise (CCW). Parameters k_b and k_f represent the switching rates from CW to CCW and from CW to CCW, respectively. Only in the CW state the CheY_p molecules can bind the motor binding sites with rate $k_+ N_{Y_p}$. Once a molecule is bound, the rate of unbinding is k_-. When the motor is bound to a molecule its state is CW, and the motor can return to the CCW state only when it is without any ligand bound [33]. This model allows us to mimic the cooperativity of the motor response, implemented by the Gillespie algorithm using programming language R 3.1.2.

Fig. S10 shows the Kullback-Leibler (KL) divergence between the probability of binding in simulations with one and two motors for different CheY_p concentrations. Note that the KL divergence is a measure for comparing probability distributions and increases for high levels of CheY_p due to competitive binding and increased fluctuations in the sensitive regime. We found that overall the divergence between the two distributions is small (< 0.09 bit).

References

[1] Endres, R. G. *et al.* Variable sizes of *Escherichia coli* chemoreceptor signaling teams. *Mol Syst Biol* **4**, 211 (2008).

[2] Shannon, C. E. A mathematical theory of communication. *Bell Syst Tech J* **27**, 379–423 (1948).

[3] Bowsher, C. G. & Swain, P. S. Environmental sensing, information transfer, and cellular decision-making. *Curr Opin Biotech* **28**, 149–155 (2014).

[4] Levchenko, A. & Nemenman, I. Cellular noise and information transmission. *Curr Opin Biotechnol* **28**, 156–164 (2014).

[5] Mc Mahon, S. S. *et al.* Information theory and signal transduction systems: From molecular information processing to network inference. *Semin Cell Dev Biol* **35C**, 98–108 (2014).

[6] Micali, G. & Endres, R. G. Bacterial chemotaxis: information processing, thermodynamics, and behavior. *Curr Opin Microbiol* **30**, 8–15 (2016).

[7] Brunel, N. & Nadal, J. P. Mutual information, Fisher information, and population coding. *Neural Comput* **10**, 1731–1757 (1998).

[8] Detwiler, P. B., Ramanathan, S., Sengupta, A. & Shraiman, B. I. Engineering aspects of enzymatic signal transduction: photoreceptors in the retina. *Biophys J* **79**, 2801–2817 (2000).

[9] Tkačik, G., Callan Jr, C. G. & Bialek, W. Information flow and optimization in transcriptional regulation. *Proc Natl Acad Sci U S A* **105**, 12265–12270 (2008).

[10] Tkačik, G., Callan Jr, C. G. & Bialek, W. Information capacity of genetic regulatory elements. *Phys Rev E* **78**, 011910 (2008).

[11] Clausznitzer, D., Micali, G., Neumann, S., Sourjik, V. & Endres, R. G. Predicting chemical environments of bacteria from receptor signaling. *PLoS Comput Biol* **10**, e1003870 (2014).

[12] Laughlin, S. A simple coding procedure enhances a neuron's information capacity. *Z Naturforsch C* **36**, 910–912 (1981).

[13] Berg, H. C. & Purcell, E. M. Physics of chemoreception. *Biophys J* **20**, 193–219 (1977).

[14] Vladimirov, N., Løvdok, L., Lebiedz, D. & Sourjik, V. Dependence of bacterial chemotaxis on gradient shape and adaptation rate. *PLoS Comput Biol* **4**, e1000242 (2008).

[15] Bernardo, J. M. Reference posterior distributions for Bayesian inference. *J R Stat Soc Ser B Stat Methodol* **41**, 113–147 (1979).

[16] Jeffreys, H. An invariant form for the prior probability in estimation problems. *P Roy Soc Lond A Mat* **186**, 453–461 (1946).

[17] Mitra, P. P. & Stark, J. B. Nonlinear limits to the information capacity of optical fibre communications. *Nature* **411**, 1027–1030 (2001).

[18] Clausznitzer, D. & Endres, R. G. Noise characteristics of the Escherichia coli rotary motor. *BMC Syst Biol* **5**, 151–151 (2011).

[19] Tu, Y. & Berg, H. C. Tandem adaptation with a common design in escherichia coli chemotaxis. *Journal of molecular biology* **423**, 782–788 (2012).

[20] Yuan, J. & Berg, H. C. Ultrasensitivity of an adaptive bacterial motor. *J Mol Biol* **425**, 1760–1764 (2013).

[21] Yuan, J., Branch, R. W., Hosu, B. G. & Berg, H. C. Adaptation at the output of the chemotaxis signalling pathway. *Nature* **484**, 233–236 (2012).

[22] Zhang, C., He, R., Zhang, R. & Yuan, J. Motor adaptive remodeling speeds up bacterial chemotactic adaptation. *Biophys J* **114**, 1225–1231 (2018).

[23] Sourjik, V. & Berg, H. C. Receptor sensitivity in bacterial chemotaxis. *Proc Natl Acad Sci U S A* **99**, 123–127 (2002).

[24] Endres, R. G. & Wingreen, N. S. Precise adaptation in bacterial chemotaxis through "assistance neighborhoods". *Proc Natl Acad Sci U S A* **103**, 13040–13044 (2006).

[25] Borkovich, K. A., Alex, L. A. & Simon, M. I. Attenuation of sensory receptor signaling by covalent modification. *Proc Natl Acad Sci U S A* **89**, 6756–6760 (1992).

[26] Dunten, P. & Koshland, D. Tuning the responsiveness of a sensory receptor via covalent modification. *Journal of Biological Chemistry* **266**, 1491–1496 (1991).

[27] Yonekawa, H. & Hayashi, H. Desensitization by covalent modification of the chemoreceptor of escherichia coli. *FEBS letters* **198**, 21–24 (1986).

[28] Sourjik, V. & Berg, H. C. Binding of the *Escherichia coli* response regulator CheY to its target measured in vivo by fluorescence resonance energy transfer. *Proc Natl Acad Sci U S A* **99**, 12669–12674 (2002).

[29] Keymer, J. E., Endres, R. G., Skoge, M., Meir, Y. & Wingreen, N. S. Chemosensing in escherichia coli: two regimes of two-state receptors. *Proc Natl Acad Sci U S A* **103**, 1786–1791 (2006).

[30] Cover, T. M. & Thomas, J. A. *Elements of Information Theory (Wiley Series in Telecommunications and Signal Processing)* (Wiley-Interscience, 1991), 99 edn.

[31] Cheong, R., Rhee, A., Wang, C. J., Nemenman, I. & Levchenko, A. Information transduction capacity of noisy biochemical signaling networks. *Science* **334**, 354–358 (2011).

[32] Cluzel, P., Surette, M. & Leibler, S. An ultrasensitive bacterial motor revealed by monitoring signaling proteins in single cells. *Science* **287**, 1652–1655 (2000).

[33] Endres, R. *Physical Principles in Sensing and Signaling: With an Introduction to Modeling in Biology* (Oxford University Press, 2013).

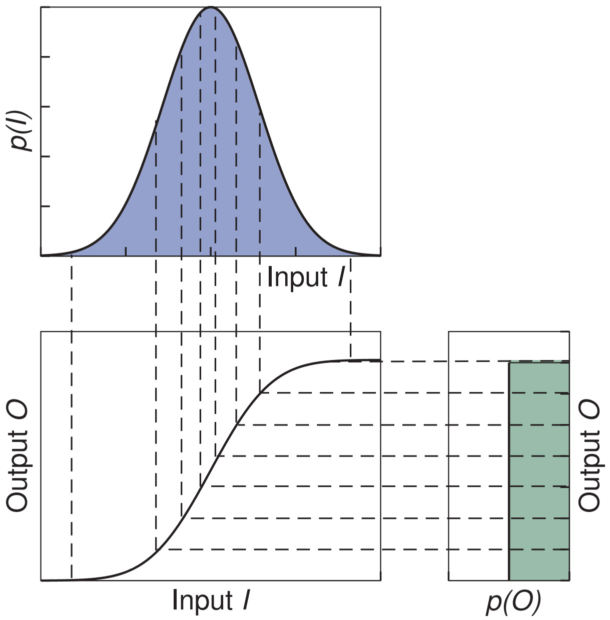

Figure S1. Optimal information transmission for uniform noise. Maximal mutual information for uniform noise leads to a uniform output distribution (bottom right). The input distribution (top) can be reconstructed given the input-output curve (middle) and vice versa, given by $p \propto \bar{y}'$ (where $\bar{y}$ is the input-output curve) [12].

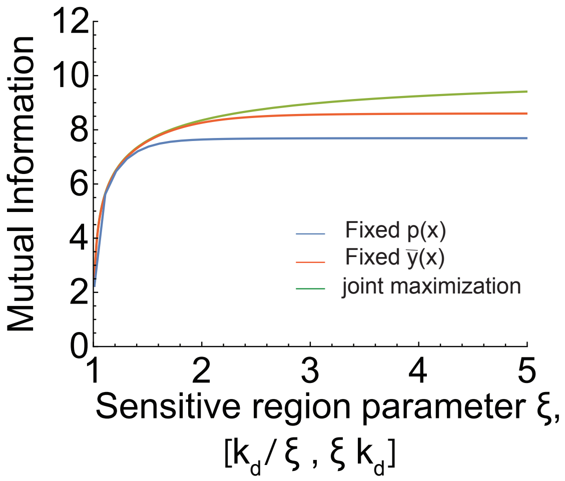

Figure S2. The role of the sensitive region on the optimal mutual information. Mutual information as a function of the size of the sensitive region. The mutual information increases and eventually saturates for the maximizations with respect to the input distribution (red line) and the input-output curve (blue line), and for the joint maximization (green line). The input-output curve is fixed (red line) or target to be a Hill function with Hill coefficient $n = 5$ and threshold constant $k_d = 0.1$ (blue line). The noise parameters are $\alpha_{1,3} = 10^{-7}$ and $\alpha_{2,4} = 10^{-8}$. Amplitude of the sensitive region is measured by the parameter ξ which define the sensitive region by $x \in [k_d/\xi, k_d\xi]$.

	Symbol	value
Receptor number in cluster	N	50
Total receptor number	N_{tot}	7000
Methylation rate	g_R	0.0069 s^{-1}
Receptor cluster size	s	0.3 μm
Receptor integration time	τ	0.1 s
Diffusion coefficient	D	300 $\mu m^2/s$
Adapted activity	A^*	0.3
Total concentration of CheY	Y_T	7.9 μM
Switching rate of receptors	$k_{1,2} = k$	50 s^{-1}
Adaptation time	ω_m	5 s

Table S1. Parameters of *E. coli* chemotaxis pathway. This table contains the parameter values and symbols used in Sec. 3.

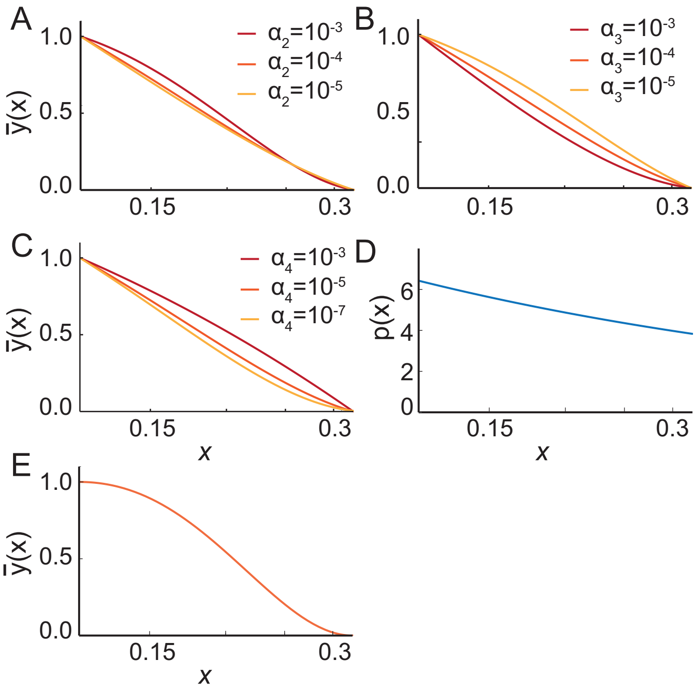

Figure S3. Noise sensitivity analysis. Dependence of the optimal input-output curve in Eq. (S34) on α_2 (A), α_3 (B), and α_4 (C). The optimal input-output curve in Eq. (S34) is independent of parameter α_1. (D) The optimal input distribution does not depend on α_{1-4}. (E) The optimal input-output curve in Eq. (S36), which has zero slope at the boundaries, coincides with the case $\alpha_{3,4} = 0$. Parameter-value changes are shown in legend, and values are $\alpha_{2,3} = 10^{-4}$ and $\alpha_{2,3} = 10^{-5}$ when not explicitly stated differently.

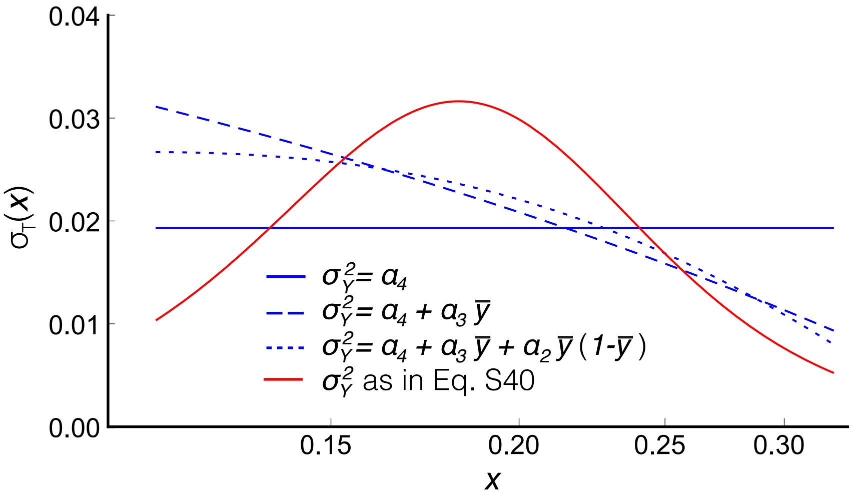

Figure S4. Total noise for optimal information transmission for different types of output noise. Eq. (S40) plotted as a function of the input for Poissonian input noise $\sigma_x^2 = \alpha_1 x$ and different types of output noise (Eq. (5) in the main text). Output noise and optimal response as explained in Sec. 1.4.1 (blue solid line), Sec. 1.4.2 (blue dashed line), Sec. 1.4.3 (blue dotted line) and Sec. 1.4.1 (red solid line). The peak in the total noise appears when the optimal response is sigmodal (cf. with Ref. [11]). Parameters α_{1-4} are rescaled to have the same total noise level (i.e. $\int \sigma_T(x)dx$ is the same for all noise types). Note that we set $\alpha_4 = \alpha_{1-3}/10$ similar to Figs. 4-5 and S9.

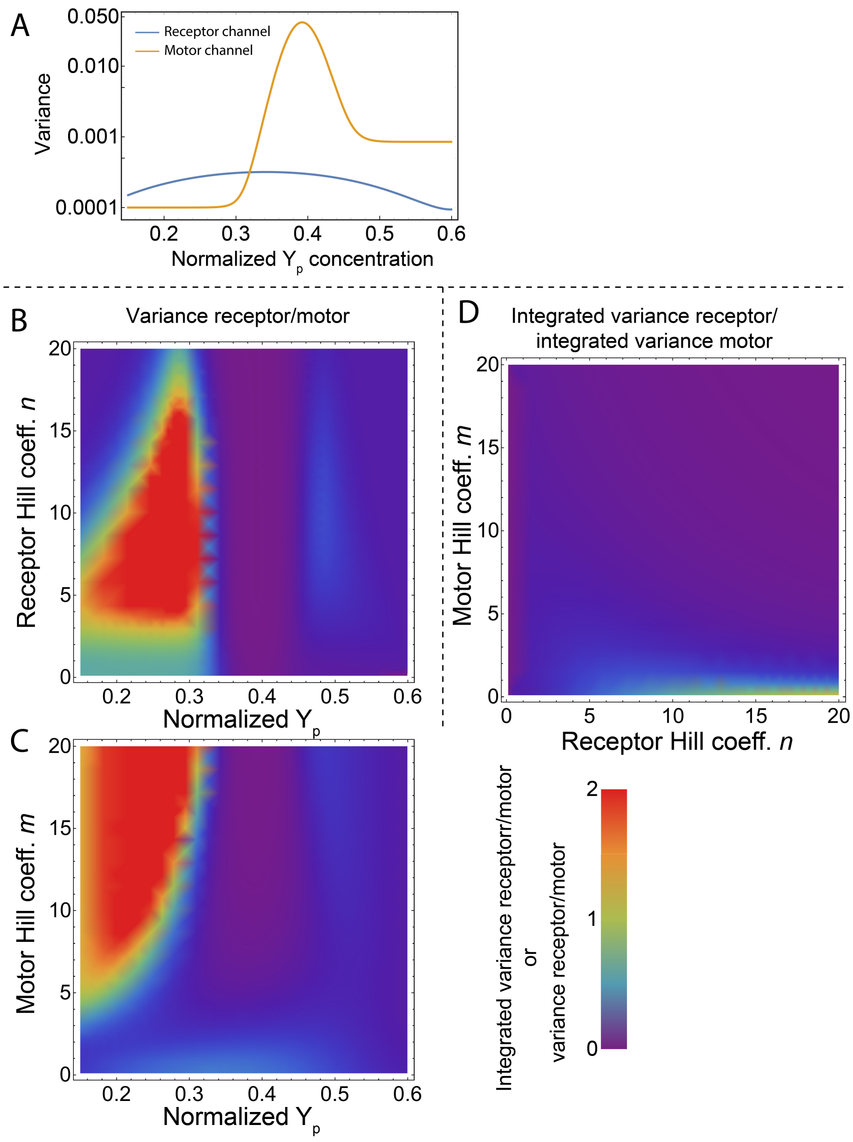

Figure S5. Comparison of the magnitude of the noise at the receptor and motor channels. (A) Noise of the receptor (blue line) and motor (yellow line) channels are plotted as a function of the normalized $\mathrm{CheY_p}$ concentration for fixed Hill coefficients $n = 5$ and $m = 20$. (B) Heatmap showing the ratio between the noise of the receptor and motor channels is plotted as function of the normalized $\mathrm{CheY_p}$ concentration and Hill coefficient n for fix $m = 20$. (C) Heatmap showing the ratio between the noise of the receptor and motor channels is plotted as function of the normalized $\mathrm{CheY_p}$ concentration and Hill coefficient m for fix $n = 5$. (D) Heatmap showing the ratio between the integrated noise of the receptor over the external concentration x and the integrated noise of the motor over the normalized $\mathrm{CheY_p}$ concentration is plotted as function of the Hill coefficients n and m. Colormap range from 0 to 2. All the other parameters (including α and β) are set as in Fig. 5 of the main text.

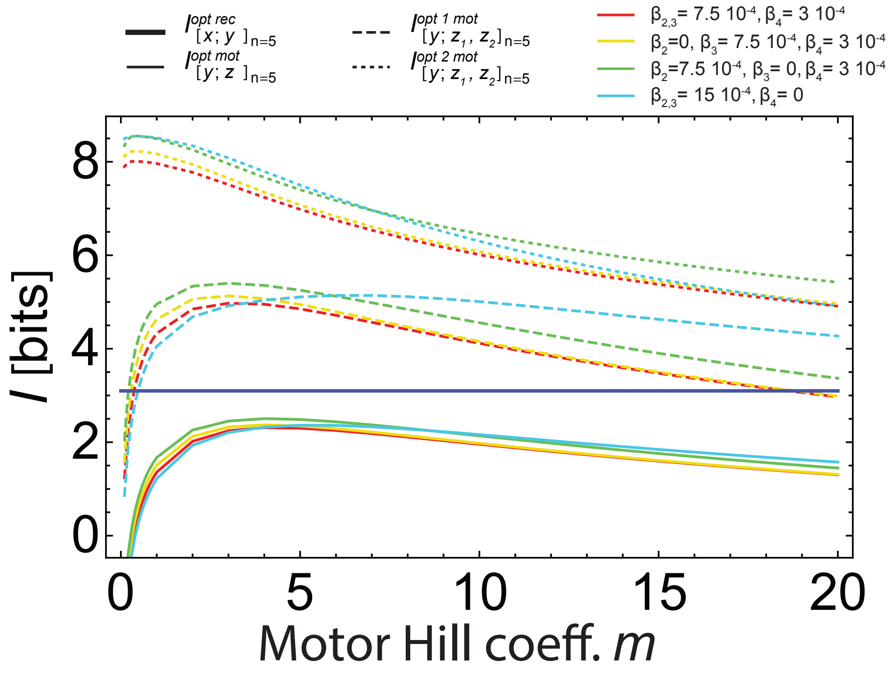

Figure S6. Robustness of the mutual information of the motor channel to changes in β values. Comparison of a single motor (solid lines), two motors where the input distribution is optimized for a single motor (*opt 1 mot*, dashed lines), and two motors where the input distribution is optimized for two motors (*opt 2 mot*, dotted lines), plotted as a function of the motor Hill coefficient m. Mutual information of the receptor channel is shown as a reference (dark blue solid line). The Hill coefficient of the receptor is kept constant $n = 5$ while a broad range of β values is explored. The red lines show the parameters used for Fig. 5 in main text. Yellow lines show the case in which the switching noise is set to zero ($\beta_2 = 0$), green lines show the case in which the sensing noise is set to zero ($\beta_3 = 0$), and light blue lines show the case in which both the switching and the sensing noise are set to zero ($\beta_{2,3} = 0$).

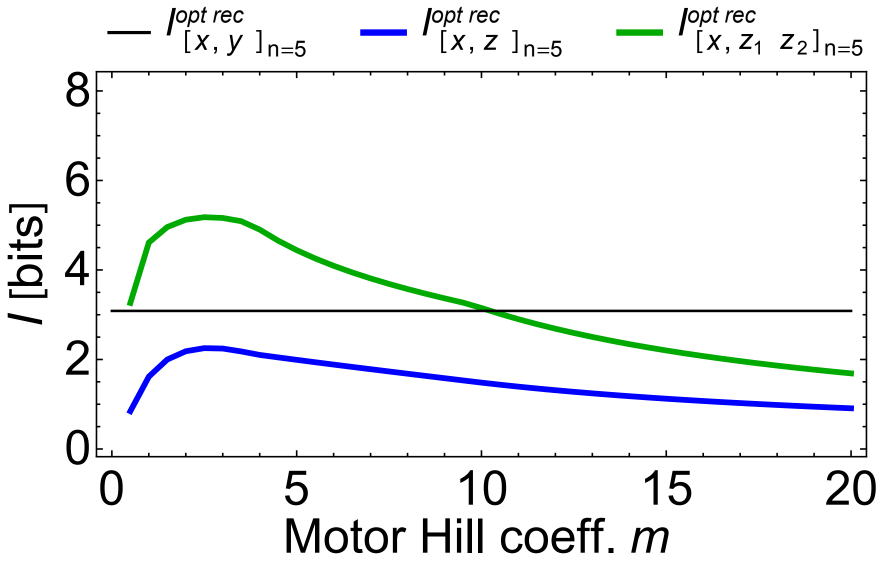

Figure S7. Mutual information between external ligand concentration and motor bias. Mutual information of the motor channel assuming optimal information transmission at the receptor is plotted as a function of the motor Hill coefficient m (blue and green lines for a single and two motors, respectively). The optimal mutual information at the receptor is plotted as reference (black line). The mutual information shows a peak at around $m \approx 3$ and then decreases for high m values similar to Fig. 5 of the main text. The mutual information of the receptor channel assuming optimal information transmission at the motor channel can similarly be investigated. Parameters as in Fig. 5 of main text.

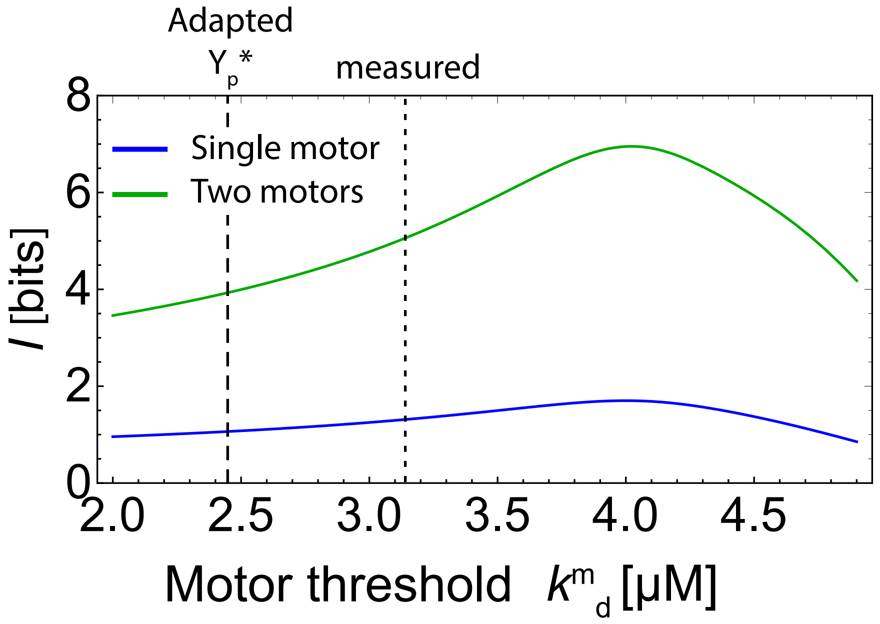

Figure S8. Mutual information as a function of the motor threshold. Maximal mutual information of the motor channel as a function of threshold k_d^m. Mutual information for a single (blue line) and two (green line) motors peaks away from the adapted $\mathrm{CheY_p}$ (Y_p^*) value (black dashed line) but close to the measured value (black dotted line).

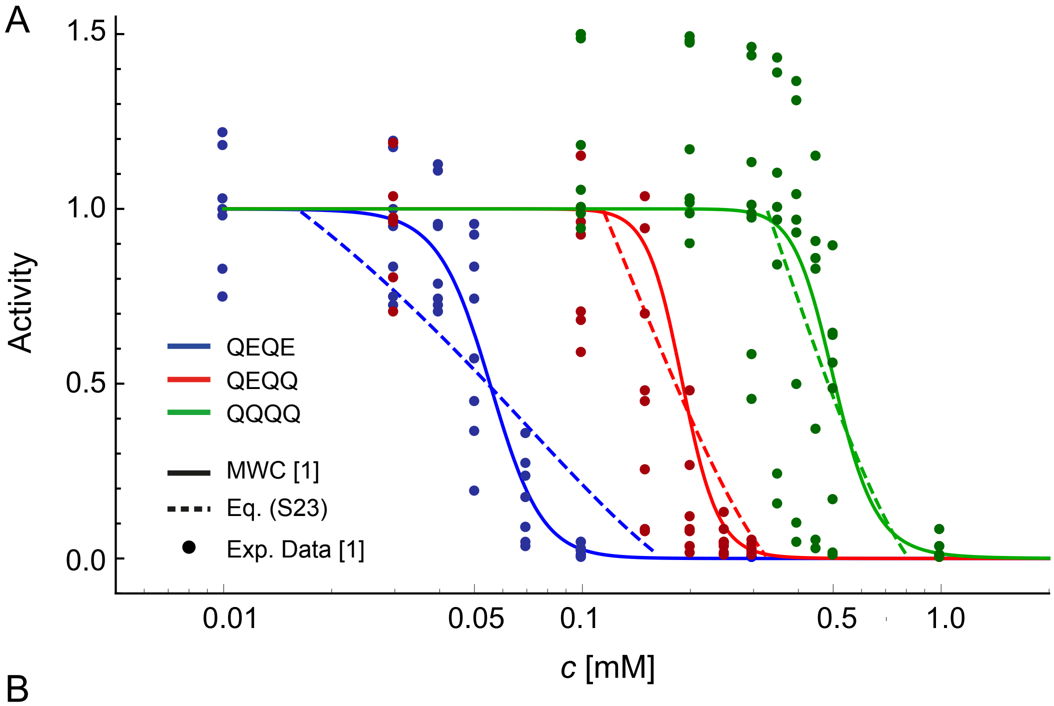

B

	Fitted Hill coefficient MWC model [1]	Fitted Hill coefficient using Eq. (S23)	Fold increase in $\mathcal{I}$ Eq. (S23) / MWC
QEQE	6.0 (5.5 - 6.6)	2.5	1.17
QEQQ	8.6 (5.1 - 12.1)	5.2	1.06
QQQQ	7.2 (4.6 - 9.8)	6.0	1.04

Figure S9. Comparison of optimal input-output curves with experimental data from [1]. (A) Receptor activity as function of MeAsp concentration for three different receptor modification levels in non-adapting mutants (mimicking different receptor methylation levels): QEQE (blue), QEQQ (red) and QQQQ (green). Comparison of the established MWC model for *E. coli* chemoreceptors (solid line) [1, 29], optimal input-output curves from Eq. (S34) (dashed line), and FRET data for low-expression level of Tar (1.4 × native) from [1] (discs). (B) Three mutants (first column), fitted Hill coefficient to the MWC model with error in estimation in brackets (second column), extracted Hill coefficients to Eq. (S34) (third column), and fold increase in mutual information, $\mathcal{I}$, of Eq. (S34) relative to the MWC model (fourth column). Parameters for the MWC model are provided in [1]. Parameters for Eq. (S34) are motivated by in Sec. 3: $\alpha_{1-3} = 10^{-4}$, and $\alpha_4 = 10^{-5}$. The sensitive regime is extracted as explained in Sec. 4.3 of *Supporting Information*.

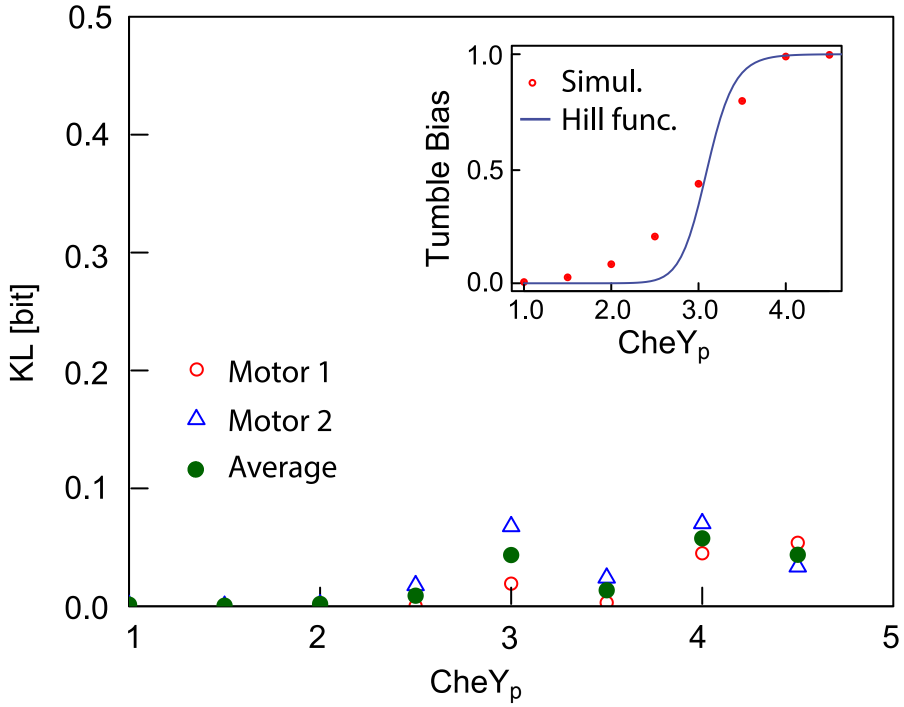

Figure S10. Kullback-Leibler (KL) divergence of the probability of CheY$_p$ bound to a motor in the case of one and two motors. The KL divergence between the probability distribution of a motor being bound in the case of a single motor and one of two motors (red circles), a single motor and the other of two motors (blue triangles), and the average of the two (green dots). The KL divergence generally remains small (< 0.09 bit) for all physiologically relevant CheY$_p$ levels. (Inset) Clockwise (CW) bias as a function of the CheY$_p$ level (red dots), in comparison to a Hill function with Hill coefficient 20 (blue solid line). The discrepancy is likely due to using 20 instead of 30 binding sites.
